# A white circular-spot pattern of iridian atrophy associated with Varicella-zoster virus and *Toxoplasma gondii* coinfection: a case report

**DOI:** 10.1186/s12886-020-01748-8

**Published:** 2020-12-07

**Authors:** Juliana Muñoz-Ortiz, Olga Lorena Rubio-Romero, Maria Cecilia Cedeño, Karla Arteaga-Rivera, Alejandra de-la-Torre

**Affiliations:** 1 Escuela Barraquer. Research Group, Escuela Superior de Oftalmología - Instituto Barraquer de América, Avenida Calle 100 # 18A – 51, Bogotá, Colombia; 2 NeURos Research Group, Escuela de Medicina y Ciencias de la Salud, Universidad del Rosario, Carrera 24 # 63 C 69, Bogotá, Colombia; 3 Fundación Valle de Lili, Valle del Cauca, Carrera 98 # 18-49, Cali, Colombia

**Keywords:** Iridian atrophy, Uveitis, Coinfection, Varicella-zoster, *Toxoplasma gondii*, Polymerase chain reaction

## Abstract

**Background:**

We report a case of white circular spots of iridian atrophy, which we will call “polka dots” pattern, as a rare ophthalmological finding associated with uveitis secondary to varicella-zoster virus and *Toxoplasma gondii* coinfection in a male patient in Bogotá, Colombia.

**Case presentation:**

We present de case of a 53-year-old Colombian male patient with a diagnosis of anterior uveitis in his left eye due to varicella-zoster virus and *Toxoplasma gondii* coinfection documented by polymerase chain reaction analysis. He presented with multiple areas of superficial white circular spots of iridian atrophy in 360º, some with deeper atrophy where the stroma fibers were visualized and only a small punctate defect of transillumination was evident. This rare pattern of iridian atrophy has not been previously described in cases of uveitis in the literature.

**Conclusions:**

This is the first case reporting the findings of superficial “polka dots” pattern iridian atrophy in 360° secondary to anterior uveitis due to the coinfection of a virus and a parasite. The identification of similar clinical cases may lead to early initiation of systemic treatment in these patients.

## Background

Infectious uveitis is the leading cause of uveitis in Colombia. Toxoplasma gondii (Tg) is the most frequent agent involved in 39.8% of these cases [1], however, ~ 7.5% of ocular toxoplasmosis cases can coexist with other infectious agents, e.g. *Mycobacterium tuberculosis,* cytomegalovirus (CMV), and varicella-zoster virus (VZV) [2].

Clinical manifestations of ocular toxoplasmosis usually include necrotizing retinochoroiditis, vitritis, optic neuritis, and less frequently, anterior hypertensive uveitis [3]. On the other hand, viral agents uveitis cause a broad clinical spectrum of clinical findings, including ocular hypertension, diffuse stellate keratic precipitates, the presence of iridian atrophy (IA) [4], retinitis, macular edema, scarring, and retinal pigment epithelium hyperplasia [5].

Iris stromal atrophy in uveitis is characterized by sectorial, spiral, or diffuse damage of the iris with transillumination defects, or rarely, massive IA with gross sphincter damage [4]. IA has been associated with iris perfusion disorders [6] and/or viral invasion of the pigment epithelium [4]. Although there are few reports of different infectious etiologies, such as toxocariasis [7] and toxoplasmosis [8] associated with iris heterochromia (Fuchs' heterochromic iridocyclitis), no reports are describing the round circumscribed pattern, which we refer to as the “polka dots” pattern, observed in our patient.

## Case presentation

**Fig. 1:**
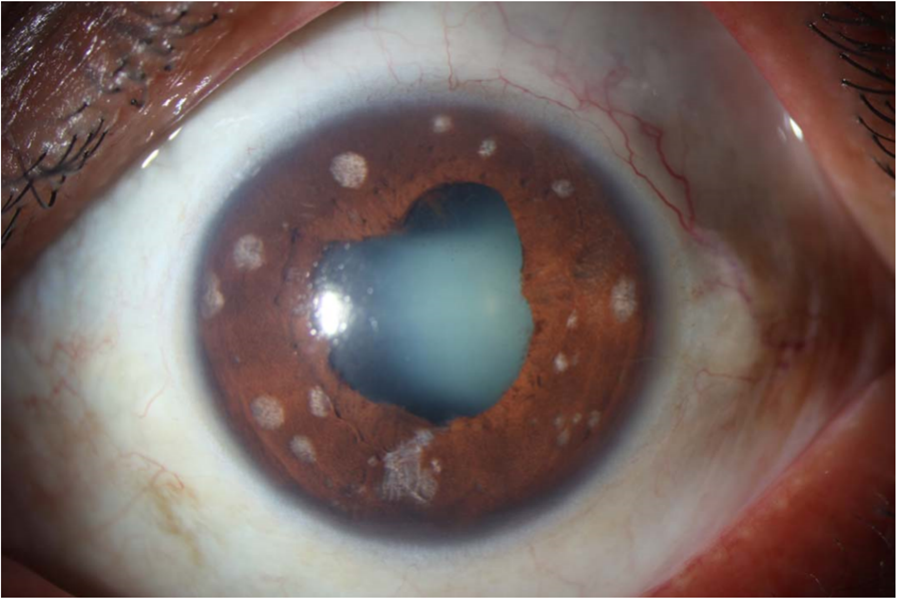
Slit-lamp photography of the anterior segment of the OS. Iris with multiple areas of superficial atrophy of a rounded aspect in 360º, posterior iris synechiae, and cataract

A 53-year-old Colombian male patient with history of ocular toxoplasmosis in his left eye (OS) with signs of posterior uveitis, a retinochoroidal lesion, IgG positive antibodies for *Tg* and negative results for other diseases such as sarcoidosis, syphilis and tuberculosis, was referred to the uveitis consultation. He was previously treated with Trimetroprim 160 mg + Sulfamethoxazole 800 mg twice daily, Clindamycin 300 mg every 6 h, and Deflazacort 30 mg per day, associated with topical therapy with 1% Prednisolone acetate in a tapering regimen. After 6 weeks the patient presented complete retinochoroiditis improvement. However, anterior chamber cellularity (Tyndall 3 +) persisted and new findings such as multiple areas of superficial circular atrophy in 360º, posterior iris synechiae (Fig. 1) and ocular hypertension were observed. Viral origin due to the presence of IA zones and hypertensive anterior uveitis, was suspected. Therefore, treatment with 1% Prednisolone acetate, 2% Dorzolamide + 0.2% Brimonidine Tartrate + 0.5% Timolol in his OS, and Acyclovir 800 mg five times a day orally for 10 days followed by Acyclovir 800 mg once a day as maintenance dose, was initiated. As a complication, the patient presented cortical opacities and posterior subcapsular cataract of the OS with surgery requirement.

**Fig. 2:**
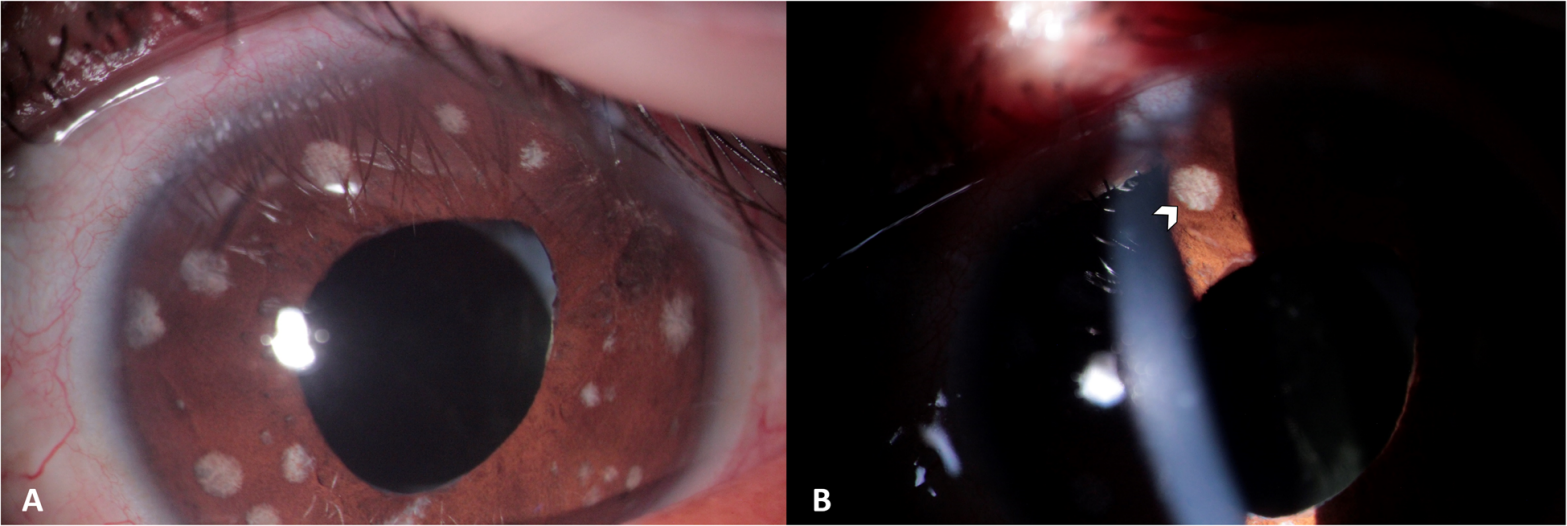
Slit-lamp photography of the anterior segment of the OS. **a.** White circular spots iridian superficial atrophy in 360º. **b.** Some with deeper atrophy where the stroma collagen fibers are visualized (arrowhead)

The patient presented reactivation of the inflammatory activity in his OS, despite treatment. Thus, an exhaustive interrogatory and exam was performed, discarding systemic autoimmune disease history and signs. At the ophthalmological evaluation, the patient presented BCVA OD 20/20 and OS 20/30. OD examination was normal. Positive findings in OS were: temporal and nasal conjunctival vessel tortuosity, 0.5 + of anterior chamber cells, increased superficial circular atrophy zones in 360º of the iris, some with deeper atrophy stroma fibers visualization (Fig. 2) and a small punctate defect of transillumination at three hours, dyscoric pupil in medium mydriasis and pseudophakia. IOP was 16/18 mmHg. Fundus observation for OD was normal and OS revealed vitreous syneresis, posterior vitreous detachment, 0.5 + vitreous cells, 0.2 optic disc excavation, and a hypopigmented retinochoroidal scar on the inferior temporal vascular arch.

Laboratories and aqueous humor PCR were requested. They showed VZV IgG 1803.5 IU / ml (positive), CMV IgG 18.6 (positive), herpes simplex virus (HSV) type 2 IgG 16.2 (positive), *Tg* IgG > 650 (positive), and all IgM tests were negative. Aqueous humor PCR analysis revealed the presence of VZV and *Tg* genetic material. The multiplex PCR qualitatively used ruled out HSV type 1, HSV type 2, Epstein-Barr virus, CMV, HHV-6 (Human Herpes Virus), HHV-7, and HHV-8 etiologies.

Treatment with oral Pyrimethamine 25 mg + Sulfadoxine 500 mg 3 tablets each 8 days for 6 weeks and Acyclovir 800 mg five times per day for 10 days followed by Acyclovir 800 mg once a day as maintenance dose, 1% Prednisolone acetate in a tapering regimen and topical tropicamide every 8 h until the resolution of inflammation in the anterior chamber was given. After four weeks of therapy, the patient denoted subjective improvement, and resolution of the intraocular inflammation was clinically observed.

## Discussion and conclusions

IA may be caused by various types of etiologies such as glaucoma, senility, trauma, surgery, and by hereditary abnormalities. In the literature reviewed, we found two cases of circular spots IA pattern, both diagnosed as vitiligo iridis. The first case was a pediatric patient and the second case was an incidental diagnosis in a 50-year-old man, both with a definite history of smallpox. The lesions were described as multiple grayish-white circular spots with a variable size between 1 to 2 mm on the anterior surface of the iris [9]. This differs from the polka dots pattern observed in our patient since the lesions of our case were bigger and delimitated, however, all cases were unilateral.

Kavitha et al. [10] in 2015 published a series of patients diagnosed with unilateral vitiligo iridis and secondary glaucoma in the same eye with a past medical history of smallpox in their childhood. These patients underwent phacoemulsification, but the iridian defects did not correlate with the atrophy that could be generated after surgery, as in the case of our patient. All three patients presented with pitted scars on their faces [10], nevertheless, we thoroughly examined our patient and found no similar lesions or areas of depigmentation.

In 1973, Spiros et al. described two cases of Afrodescendant woman with sectorial areas of whitish round superficial IA in the proximity of the collarette in both eyes and no transillumination defects. Blood smear analysis revealed anisocytosis and hyperchromic erythrocytes. Hemoglobin electrophoresis confirmed the diagnosis of sickle cell disease [11]. This differs from our patient since he was Hispanic, he did not have sickle cell disease manifestations, and the iris lesions were not around the iridian collarette but predominantly in the periphery with a smaller diameter.

There have been previously reported an association between the VZV viral load in the aqueous humor and the clinical manifestations of VZV anterior uveitis. IA finding was found to be much more severe in a high-viral load group compared with a low-viral load group of patients with VZV anterior uveitis [12].

Some theories about the pathogenesis of IA have been considered over the years. In the case of VZV, the infection seems to be associated with invasion of the pigment epithelium by the virus, an occlusive vasculitis that may produce ischemia or a neurogenic effect [13]. The vasculitis pathogenesis is secondary to a viral invasion of blood vessels from neighboring nerves or an immunogenic mechanism in response to zoster antigens resulting from viral replication in nerves in the iris [14, 15]. In these patients, fluorescein angiography of the anterior segment has shown segmental IA with the corresponding non-perfusion of radial vessels and some micro-neovascularization [6].

Histopathological studies of multiple enucleated eyes following ophthalmic herpes zoster infection showed scarring of the stroma, numerous pigment-laden macrophages, and occasionally proliferation of pigment epithelial cells. These results resemble the observations in ischemic necrosis of the iris in acute angle-closure glaucoma. Therefore, it is hypothesized that the findings described may be secondary to an acute rise in the IOP or as the result of both chronic inflammation and ischemia secondary to vasculitis [14].

Furthermore, the IOP elevation may be due to inflammation of the trabecular meshwork and the accumulation of inflammatory debris from uveitis. The IOP must be monitored at all examinations because it often increases suddenly [16]. This acute and persistent increase may lead to episodes of ischemia that cause IA, which is thought to be one of the factors contributing to the iris depigmentation shown in the case of our patient.

Both large and small arteries can be affected by VZV vasculopathy, and these may result in episodes of ischemic or hemorrhagic strokes [17]. Direct infiltration of the VZV has been found in the walls of the SNC vessels [18, 19]. In the early phase of infection, these viruses can be found in the adventitia, and in later stages, they are often found in the media and intima layers [20]. This suggests infiltration transmurally from the adventitia to the intima, possibly after extending transaxonally to the artery from the ganglion [21].

*Tg* infection usually affects the retina and choroid in the eye, however, it has also been described in syndromes with Fuchs' heterochromic uveitis etiologies [7], in which the stromal atrophy exposes substantial areas of the iris pigmented epithelium, but unlike the case of our patient without a specific “polka dots” pattern. Anterior segment findings in *Tg* infections may include granulomatous or stellate keratic precipitates, elevated IOP [22, 23], and complications, such as anterior and posterior synechiae and IA, occur in less than 5% of eyes [24].

In the case we presented here, the question arises of whether there was a summation of atrophy mechanisms of the virus and pathogen, or if the changes in the IOP caused the whitish round superficial atrophy in multiple locations in the iris.

The diagnosis of retinochoroiditis by *Tg* is clinical, however, in cases such as the previously presented, PCR is a fundamental study to determine appropriate management. The calculated percentages for sensitivity, specificity, positive predictive value, and negative predictive value of PCR in aqueous humor have been described as 91.3%, 98.8%, 98.6%, and 92.4% respectively. The multiplex PCR qualitatively used in our uveitis patients measure the genomic DNA of 8 families of herpes virus: HSV type 1, HSV type 2, VZV, Epstein-Barr virus, CMV, HHV-6, HHV-7, HHV-8, and *Tg*. [25].

In a study of 66 Colombian patients with uveitis of presumed infectious origin, PCR analysis of aqueous humor and vitreous humor samples suggested that 33.3% were ocular toxoplasmosis and 7.5% were caused by a coinfection. Of this last group, 4.5% corresponded to VZV and *Tg* co-infections [2].

In a study made with patients clinically diagnosed with active ocular toxoplasmosis, the PCR qualitative analysis in aqueous humor showed to be positive for *Tg* DNA in 37.21% of the samples and 2.33% of the peripheral blood samples [26]. Nevertheless, PCR positivity for *Tg* DNA has been found in patients with inactive retinochoroiditis scars. This is thought to happen because latent *Tg* organisms or their antigens, present in inactive retinal cysts, could be released into the vitreous, causing the uveitis, without any associated foci of active infection or retinal necrosis [27]. We consider that the patient had an active infection by both microorganisms, *Tg* and VZV, since the response to treatment was favorable by giving a new antiparasitic therapy associated with antiviral therapy.

To the best of our knowledge, this is the first study to report a case of a rare pattern consisting of multiple whitish round superficial IA, we termed a “polka dots” pattern, related to *Tg* and VZV coinfection uveitis. This manifestation may be associated with multiple physiopathological mechanisms, including ocular hypertension, local vasculitis, and inflammation in the iridian surface. We discarded several differential diagnoses, such as vitiligo, complicated cataract surgery, and hematological diseases, confirming that the cause of the patches is related to the infectious background of our patient. The identification of similar clinical cases may lead to early initiation of systemic treatment in these patients.

## Data Availability

All data generated or analyzed during this study are included in this published article and available from the corresponding author on reasonable request.

## Abbreviations

Tg: *Toxoplasma gondii*
CMV: Cytomegalovirus
VZV: Varicella-zoster virus
HHV: Human herpesvirus
IA: Iris atrophy
OS: Left eye
HSV: Herpes simplex virus
PCR: Polymerase chain reaction
IOP: Intraocular pressure
